# MicroRNA-365 alleviates morphine analgesic tolerance via the inactivation of the ERK/CREB signaling pathway by negatively targeting β-arrestin2

**DOI:** 10.1186/s12929-018-0405-9

**Published:** 2018-02-07

**Authors:** Xian-Ping Wu, Rui-Xuan She, Yan-Ping Yang, Zu-Min Xing, Han-Wen Chen, Yi-Wen Zhang

**Affiliations:** 1 Department of Anesthesiology, Shunde Hospital of Guangzhou University of Traditional Chinese Medicine, Peoples, Foshan, 528333 People’s Republic of China; 2 Department of Anesthesiology, Shunde Hospital of Southern Medical University, Foshan, 528300 People’s Republic of China

**Keywords:** microRNA-365, β-arrestin2, Morphine tolerance, Extracellular signal-regulated kinase, cAMP-response element binding protein

## Abstract

**Background:**

Morphine is widely used in clinical practice for a class of analgesic drugs, long-term use of morphine will cause the action of tolerance. MicroRNAs have been reported to be involved in morphine analgesic tolerance..

**Methods:**

Forty male SD rats were selected and randomly divided into 5 groups: the control group, morphine tolerance group, miR-365 mimic + morphine (miR-365 mimic) group, miR-365 inhibitor + morphine (miR-365 inhibitor) group and miR-365 negative control (NC) + morphine (miR-365 NC) group. After the administration of morphine at 0 d, 1 d, 3 d, 5 d and 7 d, behavioral testing was performed. A dual luciferase reporter gene assay was performed to confirm the relationship between miR-365 and β-arrestin2, RT-qPCR was used to detect miR-365, β-arrestin2, ERK and CREB mRNA expressions, western blotting was used to evaluate the protein expressions of β-arrestin2, ERK, p-ERK, CREB and p-CREB, ELISA was used to detect the contents of IL-1β, TNF-α and IL-18, while immunofluorescence staining was used to measure the GFAP expression. Intrathecal injection of mir365 significantly increased the maximal possible analgesic effect (%MPE) in morphine tolerant rats. β-arrestin2 was the target gene of miR-365.

**Results:**

The results obtained showed that when compared with the morphine tolerance group, there was an increase in miR-365 expression and a decrease in the β-arrestin2, ERK, CREB protein expressions, contents of IL-1β, TNF-α, IL-18 and GFAP expression in the miR-365 mimic group, while the miR-365 inhibitor group displayed an opposite trend.

**Conclusions:**

The results of this experiment suggest that by targeting β-arrestin2 to reduce the contents of IL-1β, TNF-α and IL-18 and by inhibiting the activation of ERK/CREB signaling pathway, miR-365 could lower morphine analgesic tolerance.

## Background

Pain is hierarchical, and its characterization plays a significant role in the diagnosis and choice of treatment and selection of analgesics and titration of the dose are guided by the clinical effects [1]. Morphine is a highly potent analgesic that provides effective pain relief. However, prolonged or repetitive use of morphine leads to decreased potency of analgesic effect. In some patients, even the maximum tolerated dose of morphine cannot achieve a sufficient analgesic effect [2, 3]. Defined as a gradual loss of drug efficacy or potency, morphine tolerance is thought to be a kind of latent hyperalgesia, where dose escalation is required to maintain the same analgesic effect, leading to an increased likelihood of side effects [4].

MicroRNAs are single-stranded non-protein-coding RNA transcripts used to regulate gene expression. More than 60% of mammalian mRNA transcripts control thousands of gene networks, many of which relate to the development and function of nervous system [5]. As an onco-miR, miR-365 is highly expressed in both cells and clinical specimens of malignancies [6], and proved to be involved in cell differentiation, proliferation and apoptosis of cells including lung cancer cells and endothelial cells [7, 8].Previous data showed that miR-365 was robustly decreased in the spinal cord after chronic morphine administration, during which, the overexpression of miR-365 prevents and reverses morphine tolerance, and increased expression of miR-365 will result in a decreased expression of β-arrestin 2 protein [4].

The β-arrestin proteins, containing β-arrestin 1 and 2, are also known as cytosolic adapter proteins and can be found in both the cytosol and the nucleus [9, 10]. According to reports, morphine efficacy provides a useful strategy for treatment of chronic intractable pain and morphine tolerance in vivo and can be improved by suppression of β-arrestin 2 in the brain with specific antigene RNAs (agRNAs) [11–13].

Based on these findings, it was noted that β-arrestin 2 plays an important role in morphine tolerance. Therefore, the following study was conducted to explore the effects of miR-365 on morphine analgesic tolerance by regulating β-arrestin2 in a rat model.

## Methods

### Study subjects

Forty healthy male Sprague-Dawley (SD) rats (10–12 weeks old, 280 ± 20 g) provided by the Laboratory Animal Center of The Third Hospital of Hebei Medical University were used for the experiment. The rats were raised under a 12-h rhythm (12 h of light and 12 h of darkness) at a constant temperature of 20 °C and humidity of 50%. They were randomly divided into the control (injected with saline), morphine tolerance, miR-365 mimic + morphine (miR-365 mimic), miR-365 inhibitor + morphine (miR-365 inhibitor) and miR-365 negative control (NC) + morphine (miR-365 NC) groups, each group containing 8 rats. Lentiviral packaging products, including miR-365 mimics, miR-365 inhibitors and miR-365 NC were purchased from Shanghai Genechem Co., Ltd. (Shanghai, China). The Lentivirus titer was 3 × 10^8^ TU/mL. All procedures were strictly performed in accordance with the regulations of animal rights.

### Intrathecal catheterization of rats

Anesthesia was administered through an intraperitoneal injection of 2% pentobarbital sodium (11,715, 60 mg·kg^− 1^, Sigma Corp., San Francisco, CA, USA) in rats placed in a supine position. Hair from the neck and lumbar regions was removed and covered with aseptic hole-towel after iodine disinfection. An incision (1 cm) was made on the iliac crest L 5–6 space, and the intervertebral space was punctured with a thick needle. A polyethylene (PE)-10 pipe (Becton, Dickinson and Company, NJ, USA) was put into the subarachnoid space (3.5 cm) and was fixed on the subcutaneous fascia, resulting in an outflow of clear cerebrospinal fluid. The PE pipe was placed and firmly fixed 2-3 cm outside of the neck incision, preventing the catheter from folding. Folded. Physiological saline (20 μL) was used to wash the catheter and tissue forceps were used to close the catheter to prevent leakage. The rats were fed eight hours later in a warm and quiet environment with lower lighting. After a day, 20 μL of 2% Lidocaine (L5783, Sigma Corp., San Francisco, CA, USA) was slowly injected into the rats. The rats weren’t able to lift their left and right hind limbs 10 s after the injection and showed no retraction reflex during acupuncture. The left and right hind limb movements were recovered 10 min later, indicating catheter was rightly placed and the model was successfully established.

On the second day, behavioral tests were conducted for familiarization training and basic value measurement. On the third day, the rats in the control group were injected with 10 μL physiological saline at 8:00–9:00 a.m. and 16:00–17:00 p.m. every day for seven days. The rats in the morphine tolerance group were injected with 10 μL morphine (10 mg/mL morphine injection liquid diluted with physiological saline to 1 mg/mL, Qinghai Pharmaceutical Co., Ltd., Qinghai, China) in the morning and in the afternoon for seven days (rats were injected with 10 μL physiological saline 30 min before the morphine administration). The rats in the miR-365 mimic group were injected with 10 μL morphine in the morning and afternoon every day for seven days in addition to being injected with 10 μL of miR-365 mimics (5’-UAAUGCCCCUAAAAAUCCUUAU-3′) before morphine administration in the morning. In the miR-365 inhibitor group, the rats were injected with 10 μL morphine in the morning and afternoon every day for seven days as well as being administered with 10 μL of miR-365 inhibitors (5’-AUAAGGAUUUUUAGGGGCAUUA-3′) before morphine administration in the morning. Finally, in the miR-365 NC group, the rats were injected with 10 μL morphine in the morning and afternoon every day for seven days and were injected with 10 μL of miR-365 NC (5’-UUCUCGAACGUGUCACGUUUU-3′) before morphine administration in the morning. Compared with the control group, the analgesic effect of the morphine tolerance group was markedly reduced after seven days, indicating that the model for chronic morphine tolerance had been successfully established [4, 14].

### Lentivirus vector construction

The siRNA sequences of β-arrestin2 were designed with BLAST software according to the cDNA sequence of β-arrestin2 in GenBank. Based on the request of PLKO.1-sP6-GFP restriction site of shRNA expression plasmids, cDNA sequence of shRNA (GCTAATGCTTGGCGTATTACC) was designed and synthesized by Shanghai Genechem Co., Ltd. oligo DNA was then diluted to 200 μmol/L with sterile water. Afterwards, the 20 μL reaction system consisted of 5 μL forward and 5 μL reverse oligo DNA, 10 × Oligo Annealing Buffer, and 8 μL sterile water. And the reaction condition was: denaturation at 95 °C for 5 min and anneal at 70 °C for 10 min. Samples were taken out and dropped into water bath cauldron until they cooled to room temperature. Then they were diluted a 100 times with sterile water without RNA enzyme, 50 times with 10 × Oligo Annealing Buffer, and then diluted to 10 nmol/L. The double stranded oligomer obtained was connected with PLKO.1-sP6-GFP. The reaction system comprised of 5 μL 2 × ligase buffer, 2 μL double stranded oligomer, 1 μL PLKO.1-sP6-GFP expression vector, 1 μL T4 ligase (1 U/μL) and 1 μL sterile water. The above samples were mixed and incubated at 37 °C for 3 h and the β-arrestin2 shRNA expression plasmid was constructed. Then the samples were transferred into *Escherichia coli* DH5 alpha competent cells, cultured in culture plate containing ampicillin and incubated at 37 °C overnight. The following night, selected colonies were cultured and identified with PCR amplification instrument (AG22331, Eppendorf, Hamburg, Germany). The primer was T7 (5’-GGGCAGGAAGAGGGCCTAT-3′) and Sp6 (5’-TACGATTTAGGTGACACTATAG-3′). GeneGenius was purchased from GeneGenius, Syngene, Cambridge, UK. The correctly sequenced shRNAs were then inserted into the vector plasmids. Lentivirus titer was 5 × 10^8^ TU/mL and each rat was injected with 10 μL of lentivirus through an intrathecal injection.

### Paw withdrawal thermal latency (PWTL)

The rats were tested for PWTL after morphine administration at 0 d, 1 d, 3 d, 5 d and 7 d. Under constant temperature (around 25 °C), the rats were put into glass test cases (20 cm × 20 cm × 20 cm) for 15–20 min until the rats were calm and at rest. A foot radiant heat pain tester (2390, American IITC Life Science Inc., USA) was preheated with an infrared light source for about 5 min. The light intensity was adjusted at 10 s until the normal rats’ paws appeared to have a withdrawal reaction and accumulation points were adjusted at the bottom of test cases. Infrared light source was used in the test cases to irradiate the rats’ left hind paws when they became quiet. PWTL was recorded when pain and withdrawal reactions were observed in rats. The single infrared light irradiation did not exceed 20 s (cutoff time), and the same part of each rat was repeatedly measured 6 times with 5 min intervals. The maximum and the minimum measurements were eliminated, and the average measurements were calculated as the basal PWTL latency. The PWTL of rats was determined 30 min after morphine administration. The percentage of the maximal possible analgesic effect (%MPE) in 30 min was calculated after all the measurements were recorded: (%MPE = (basal PWTL latency after administration - basal PWTL latency) / (cutoff time- basal PWTL latency) × 100% [4, 15, 16].

### Dual luciferase reporter gene assay

The pMIR-REPORT-arr-3 U (GGGCATT was the binding sites of miR-365 in sequence) and pMIR-REPORT-arr-3 U-Del (GT was the mutant site of GGGCATT), restriction sites of Xhol I and Not I were put into the 5’end and 3’end of target fragments respectively, and were then converted into DH5α, and the plasmids were extracted for sequencing (Shanghai Invitrogen Biotechnology Corporation, Shanghai, China). The HEK293 cells (human embryonic kidney cells, presented by Laboratory of Cell Biology of the People’s Liberation Army General Hospital) were put into the constant temperature cell culture box (37 °C and 5% CO_2_) after recovery. Then the cells were passed when 80% confluence was reached and the cells in logarithmic growth phase were transfected. Lipofectamine™ 2000 (11668-019, Invitrogen Inc., Carlsbad, CA, USA) was used for transfection. The cells were independently transfected with pMIR-REPORT-arr-3 U and miR-365 NC, pMIR-REPORT-arr-3 U and miR-365 mimics, pMIR-REPORT-arr-3 U and miR365 inhibitors, pMIR-REPORT-arr-3 U-Del and miR365 mimics, pMIR-REPORT-arr-3 U-Del and miR-365 inhibitors, and pMIR-REPORT-arr-3 U-Del and miR365 NC. Four wells were set for each transfection. Forty-eight hours after transfection, F represented the measurements for reactions after adding firefly luciferase substrate and R represented the measurements for reactions after adding renilla luciferase substrate. The F/R value was the relative luciferase activity. CO_2_ cell culture box and multifunctional microplate instrument were bought from Thermo Fisher Scientific Corporation (VL0000D0, San Jose, California, USA). The dual luciferase reporter gene assay kit was bought from Promega Corp (E1910, Madison, Wisconsin, USA) [4, 17].

### Reverse transcription quantitative polymerase chain reaction (RT-qPCR)

The anesthesia of rats was carried out by injection of 2% pentobarbital sodium (60 mg·kg^− 1^) and lumbar enlargement region in the spinal cord of the rats was collected in an ice bath. Then 0.9% saline was used to wash the bloodstains on the surface of spinal cord followed by fast freezing in liquid nitrogen and transferring into a refrigerator at − 80 °C. The RNA was extracted from the spinal cord using Trizol. The optical density (OD) 260/280 values of each RNA sample were tested and the RNA concentrations were calculated. According to the directions of the miR-365 detection kit (LK-0101A, Shanghai Novland BioPharma, Shanghai, China), reaction conditions comprised of 3 min at 94 °C, 1 cycle, 20 s at 94 °C and 40 s at 62 °C, 40 cycles. The mRNA expressions of β-arrestin2, ERK and CREB were detected in accordance with the Reverse Transcription System (A3500, Promega Corp., Madison, Wisconsin, USA). According to the gene sequences published in GenBank database, Primer 5.0 was used to design primers as followed (Table 1), and primers were synthesized by Shanghai Sangon Biological Engineering Technology & Services Co., Ltd. The two-step reaction conditions of RT-qPCR (SYBR GREEN methods) consisted of degeneration at 95 °C for 15 min, 1 cycle, 30 s at 95 °C and 1 min at 58 °C, 40 cycles. Glyceraldehyde-3-phosphate dehydrogenase (GAPDH) was used as the internal control gene. The standard of control group was set as 1 and the cycle threshold (Ct) values (the inflection point of an amplification curve) were obtained for the target genes of each sample. The relative expression of target genes was calculated according to the relative quantification (RQ) = 2^-ΔΔCt^, and RQ values were applied for statistical analysis. Fluorescence quantitative PCR (iQ5) was bought from Bio-Rad Laboratories, Inc. (CA, USA) [4, 18].

**Table 1:** Primer sequences for RT-qPCR

Primer	Forward (5′-3′)	Reverse (5′-3′)
miR-365	CGTAATGCCCCTAAAAAT	GTGCAGGGTCCGAGGT
U6	CTCGCTTCGGCAGCACA	AACGCTTCACGAATTTGCGT
β-arrestin2	CCAGGGTCTTCAAGAAGTC	TTGCCCAAGTACACGGT
ERK	CTCTGTCATTGCCACCA	ATCCACTCTCCATCTCCAT
CREB	TACCCAGGGAGGAGCAATACA	GGTGCTGTGCGAATCTGGTAT
GAPDH	CCGAGGGCCCACTAAAGG	TGCTGTTGAAGTCACAGGAGACA

*RT-qPCR* Reverse transcription quantitative polymerase chain reaction, *ERK* Extracellular signal-regulated kinase, *CREB* cAMP-response element binding protein, *GAPDH* Glyceraldehyde-3-phosphate dehydrogenase

### Western blotting

The spinal cord tissues of rats were isolated and the protein concentrations were determined using a bicin-choninic acid (BCA) protein assay kit (P0009, Beyotime Biotechnology Co., Shanghai, China). The 5 × buffer (P0015L, Beyotime Biotechnology Co., Shanghai, China) was added to the sample and boiled for 10 min at 95 °C and the 30 μg samples were put into each hole. Polyacrylamide gel (10%) electrophoresis (Wuhan Boster Biological Technology Ltd., Wuhan, China) was performed to separate the protein sample, with the electrophoretic voltage from 80 V to 120 V, wet transferred, membrane voltage 100 mv, and 45–70 min. The protein sample was transferred to polyvinylidene fluoride (PVDF) membrane (E578, AMRESCO, Washington, USA) and was blocked with 5% bovine serum albumin (BSA) (Beijing Huamei Biological Engineering Co., LTD, Beijing, China) at room temperature for one hour. Primary antibodies GAPDH (ab37168, 1 μg/mL, Abcam, Cambridge, UK), β-arrestin2 (ab54790, 1 μg/mL; Abcam, Cambridge, UK), extracellular signal-regulated kinase (ERK) (ab54230, 1 μg/mL, Abcam, Cambridge, UK), phosphorylated (p)-ERK (ab156919, 1 μg/mL, Abcam, Cambridge, UK), cAMP-response element binding protein (CREB) (ab32515, 1: 1000, Abcam, Cambridge, UK) and p-CREB (ab32096, 1: 5000, Abcam, Cambridge, UK) diluted with Tris-buffered saline with Tween-20 (TBST) (Beijing Bioco Laibo Technology Co., Ltd., Beijing, China) were added and placed overnight at 4 °C. The membranes were washed 3 times with TBST, for 5 min each, and then corresponding second antibodies were added for reaction for 2 h at 37 °C. The membranes were washed again and A, B chromogenic agents were added (W1001, 1: 1; Promega Corp., Madison, Wisconsin, USA) for visualization for 1 min at room temperature. After visualization, samples were wrapped in plastic wrap and transferred into the dark room. The processes of developing and fixing were done after the exposure of X-ray film. Band pattern analysis was done using the Gel-Pro analyzer 4.0 image analysis software, and the expression of target protein was reflected by the ratio of target protein gray values to GAPDH gray values. Primary and secondary antibodies were bought from Abcam Inc. (Cambridge, MA, USA). Redirector decolorization Table (ZD-9500) was purchased from Taicang Hualida Experimental Equipment Co., Ltd. (Jiangsu, China). Vertical electrophoresis tank was bought from Bio-Rad Laboratories, Hercules (CA, USA) [4, 18].

### Enzyme-linked immunosorbent assay (ELISA)

A total of 100 mg of spinal cord tissue was cut, homogenized and centrifuged at 4000 rpm for 10 min at 4 °C, and the supernatant was preserved at − 80 °C. The protein of IL-1β, TNFα and IL-18 in tissues were assayed in accordance with the instructions of the corresponding ELISA kit (Shenzhen Jingmei Electronic Technology Co., Ltd., Shenzhen, China) respectively. The ten standard holes were set on the enzyme labeled plate (including 2 blank control holes without samples and conjugate reagent). The standard substance was used to draw the standard curve through gradient dilution. After the addition and mixture of the samples, the plate was sealed and incubated for 30 min at 37 °C. Then the liquid in the hole was removed, and the washing liquid was added. After 30 s, the liquid was discarded. The aforementioned process was repeated 5 times. Afterwards, 50 μL ELISA reagent was added and incubated at 37 °C for 30 min. Then liquid in the hole was removed, and washing liquid was added. After 30 s, the liquid was discarded; repeated 5 times. The 50 μL chromogenic agent A was added into each hole and 50 μL chromogenic agent B was added subsequently. Then samples were mixed and incubated for 15 min at 37 °C, and 50 μL stop buffer was added in the end. The blank control hole was set as zero and the OD value (at 450 nm) of each hole was measured within 15 min. Microplate reader was purchased from Bio-Rad Laboratories (xMark™, BioRad, Hercules, CA, USA) [16, 19].

### Immunofluorescence staining

The paraffin-embedded sections of spinal cord tissues in rats were heated at 60 °C for 1 h and were dewaxed twice with xylene; ethanol and distilled water were used for hydration. Afterwards, the tissues were boiled in a citrate buffer solution (pH = 6.0) for 15 min for sterilization and inactivation of endogenous peroxidase. The appropriate amount of the primary antibody, rabbit anti-mouse glial fibrillary acidic protein (GFAP) (1 μg/mL, HPA056030, Sigma, San Francisco, CA, USA) was added after dilution, for a reaction overnight at 4 °C and was then washed with phosphate buffer saline (PBS) 3 times, each time for 5 min. The secondary antibody, goat anti-rabbit (1: 200, Beijing Zhongshan GoldenBridge Biotechnology Co., Ltd., Beijing, China) after dilution corresponding to primary antibodies was added in the dark for incubation for 2 h at 37 °C, and then the samples were washed with PBS three times, each time for 5 min. Anti-quenched fluorescence Mounting Medium was used for mounting in the dark and the samples were kept away from light, observed and photographed under the confocal laser scanning microscope in the dark. Positive expression area of GFAP and total tissue area in the immunofluorescence images were analyzed and calculated with the Image Pro Plus 6.0 software. The rate of GFAP positive expression area was obtained. The rate of positive expression area = area of positive expression tissue of local cerebral injury/total area of local cerebral injury × 100% [4].

### Statistical analysis

All data was analyzed by SPSS 20.0 software (IBM Corp. Armonk, NY, USA) and the measurement data was expressed as mean ± standard deviation (SD). Comparisons between two groups were analyzed using *t*-test and comparisons among multiple groups using one-way analysis of variance (ANOVA). *P* < 0.05 was indicative of statistical significance.

## Results

### Overexpression of miR 365 elevates %MPE in morphine tolerance rats

**Fig. 1:**
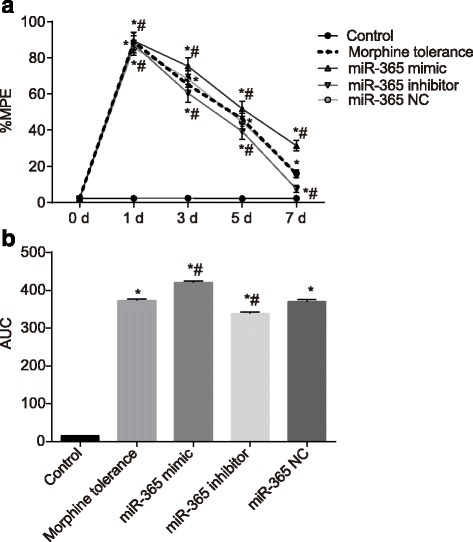
The %MPE of rats in each group after administration at 0 d, 1 d, 3 d, 5 d and 7 d. Note: A, line graph of %MPE in each group; B, AUC analyses of %MPE in each group; %MPE, percentage of the maximal possible analgesic effect; *N* = 8; ^*^, *P* < 0.05 compared with the control group; ^#^, *P* < 0.05 compared with the morphine tolerance group; AUC, area under the curve; NC, negative control

PWTL was used to detect %MPE at 30 min. According to the results of behavioral tests, the %MPE of rats showed no significant difference among five groups after they were administrated at 0 d (the next day of catheterization) (*P* > 0.05). The %MPE of the control group also showed no significant difference at 1 d, 3 d, 5 d and 7 d (*P* > 0.05). Compared with the control group, the other groups all produced maximal analgesic effect after they were administrated (*P* < 0.05) and the %MPE showed differences 3 days after administration. Compared to the morphine tolerance group, the %MPE showed no difference in the miR-365 NC group (*P* > 0.05). Compared with the morphine tolerance and miR-365 NC groups, the %MPE showed significant difference in the miR-365 mimic and miR-365 inhibitor groups. The %MPE in the miR-365 NC group was much stronger than that of the morphine tolerance in the miR-365 NC groups (*P <* 0.05), while the %MPE in the miR-365 inhibitor group was weaker than the morphine tolerance in the miR-365 NC groups (*P <* 0.05). The morphine tolerance in the miR-365 NC group began to appear 3 days after administration, and apparent tolerance was observed 7 days in. The analgesic effect decreased gradually over time. The analgesic effect of the miR-365 mimic group and the miR-365 inhibitor group also decreased gradually over time (Fig. 1). The above findings indicated that miR365 overexpression might increase the %MPE.

### β-arrestin2 is a target gene of miR-365

**Fig. 2:**
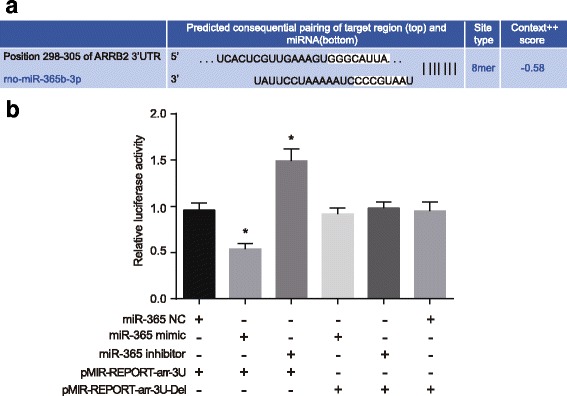
Dual luciferase reporter gene assay illustrating that miR-365 targeted β-arrestin2. Note: A, binding sites for miR-365 and 3’ UTR of β-arrestin2; B, dual luciferase reporter gene assay for miR-365 and 3’ UTR of β-arrestin2; *N* = 3; ^*^, *P* < 0.05 compared with the co-transfected group of pMIR-REPORT-arr-3 U and miR-365 NC; NC, negative control

Dual luciferase reporter gene assay was employed to verify the targeting relationship between miR-365 and β-arrestin2. The results of bioinformatics showed the matching sequence of miR-365 and 3’ UTR in β-arrestin2, as shown in Fig. 2-A. The activity of luciferase in HEK293 cells that were transfected with pMIR-REPORT-arr-3 U and miR-365 mimics decreased significantly compared to those co-transfected with pMIR-REPORT-arr-3 U and miR-365 NC (*P* < 0.05), while the activity of luciferase in HEK293 cells transfected with pMIR-REPORT-arr-3 U and miR-365 inhibitors increased significantly (*P* < 0.05). The activity of luciferase did not change notably in HEK293 cells that were transfected with pMIR-REPORT-arr-3 U-Del and miR-365 mimics, pMIR-REPORT-arr-3 U-Del and miR-365 inhibitors, and pMIR-REPORT-arr-3 U-Del and miR-365 NC, as shown in Fig. 2-B. It can be concluded that β-arrestin2 might be a target gene of miR-365.

### The overexpression of miR-365 decreases the mRNA expressions of β-arrestin2, ERK and CREB

**Fig. 3:**
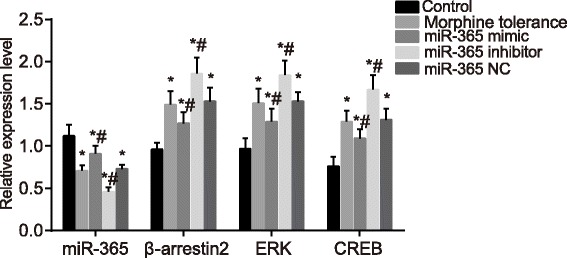
Expressions of miR-365, β-arrestin2, ERK and CREB mRNA of rats in spinal cord tissues in each group after administration at day 7. Note: N = 8; ^*^, *P* < 0.05 compared with the control group; ^#^, *P* < 0.05 compared with the morphine tolerance group; ERK, extracellular signal-regulated kinase; CREB, cAMP-response element binding protein; NC, negative control

RT-qPCR was used to detect miR-365 expression and the mRNA expressions of β-arrestin2, ERK and CREB. Compared with the control group, the miR-365 expression in the morphine tolerance, miR-365 mimic, miR-365 inhibitor and miR-365 NC groups decreased, while the mRNA expressions of β-arrestin2, ERK, and CREB increased (all *P* < 0.05). Compared with the morphine tolerance group, the miR-365 expression in the miR-365 mimic group increased, while the mRNA expressions of β-arrestin2, ERK and CREB decreased (all *P* < 0.05). The miR-365 expression in the miR-365 inhibitor group decreased, while the mRNA expressions of β-arrestin2, ERK and CREB increased (all *P* < 0.05). The miR-365 expressions in the miR-365 NC group, β-arrestin2, ERK and CREB mRNA showed no significant difference (*P* > 0.05), as shown in Fig. 3. It can be concluded that miR-365 overexpression might reduce the mRNA expressions of β-arrestin2, ERK and CREB.

### Overexpressed miR-365 inhibits the activation of ERK/CREB signaling pathway

**Fig. 4:**
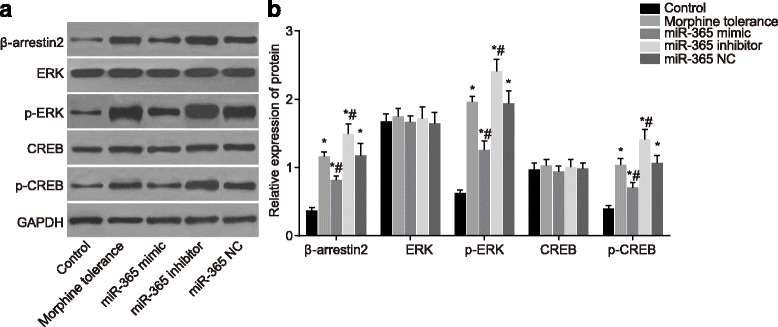
Protein expressions of β-arrestin2, ERK, p-ERK, CREB and p-CREB of rats in spinal cord tissues in each group after administration at day 7. Note: A, western blotting image of β-arrestin2, ERK, p-ERK, CREB and p-CREB; B, relative expressions of β-arrestin2, ERK, p-ERK, CREB and p-CREB; N = 8; ^*^, *P* < 0.05 compared with the control group; ^#^, *P* < 0.05 compared with the morphine tolerance group; ERK, extracellular signal-regulated kinase; CREB, cAMP-response element binding protein; NC, negative control

Western blotting was used to detect the protein expressions of β-arrestin2, ERK and CREB. According to the results, the ERK and CREB protein expressions in five groups had no statistical difference (*P* > 0.05). Compared with the control group, the protein expressions of β-arrestin2, p-ERK and p-CREB in the morphine tolerance, miR-365 mimic, miR-365 inhibitor and miR-365 NC groups increased (*P* < 0.05). Compared with the morphine tolerance group, the protein expressions of β-arrestin2, p-ERK and p-CREB showed no significant changes in the miR-365 NC group (*P* > 0.05). Compared with the morphine tolerance and miR-365 NC groups, the protein expressions of β-arrestin2, p-ERK and p-CREB in the miR-365 mimic group decreased (*P* < 0.05), while the protein expressions of β-arrestin2, p-ERK and p-CREB in the miR-365 inhibitor group increased (*P* < 0.05) (Fig. 4). We can conclude that miR-365 overexpression might reduce the protein expressions of β-arrestin2, ERK and CREB, thus inactivating the ERK/CREB signaling pathway.

### Overexpressed miR-365 decreases contents of IL-1β, TNF-α and IL-18

**Fig. 5:**
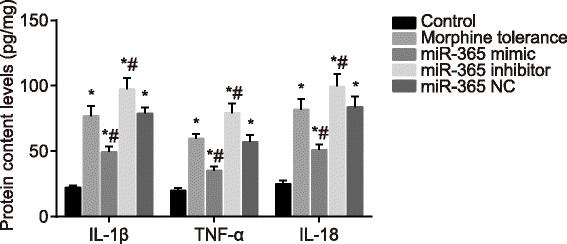
Contents of IL-1β, TNF-α and IL-18 of rats in spinal cord tissues in each group after administration at day 7. Note: N = 8; ^*^, *P* < 0.05 compared with the control group; ^#^, *P* < 0.05 compared with the morphine tolerance group; IL-1β, interleukin 1 beta; TNF-α, tumor necrosis factor alpha; IL-18, interleukin 18; NC, negative control

ELISA was adopted to evaluate the contents of IL-1β, TNF-α and IL-18 of rats in each group. Compared with the control group, the contents of IL-1β, TNF-α and IL-18 in the morphine tolerance, miR-365 mimic, miR-365 inhibitor and miR-365 NC groups markedly increased (all *P* < 0.05). Compared with the morphine tolerance group, the contents of IL-1β, TNF-α and IL-18 in the miR-365 NC group showed no significant differences (*P* > 0.05); the contents of IL-1β, TNF-α and IL-18 in the miR-365 mimic group decreased (*P* < 0.05), while the content of IL-1β, TNF-α and IL-18 in the miR-365 inhibitor group increased (*P* < 0.05) (Fig. 5). The above results implied that miR-365 overexpression might decline the contents of IL-1β, TNF-α and IL-18.

### Overexpressed miR-365 represses the activation of astrocyte

**Fig. 6:**
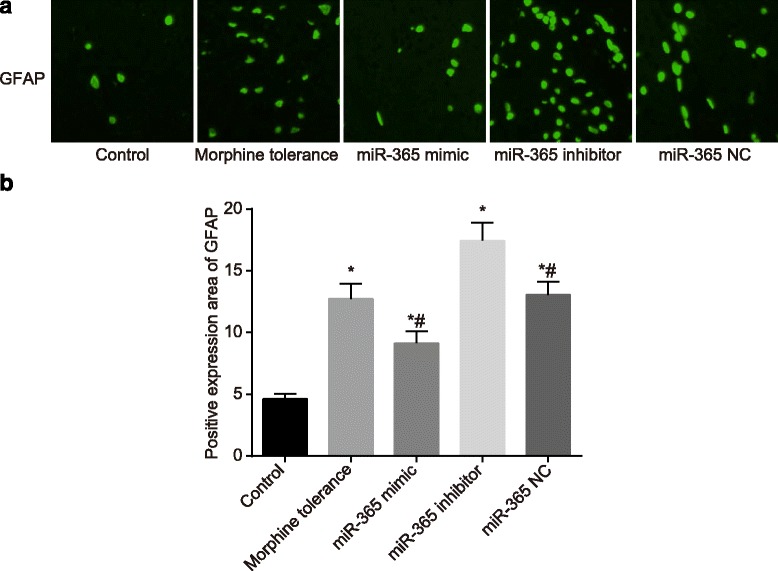
GFAP protein expressions of rats in spinal cord tissues in each group after administration at day 7. Note: A, immunofluorescence staining of GFAP in each group; B, rate of GFAP positive expression area in each group; scale bar, 200 μm; N = 8; ^*^, *P* < 0.05 compared with the control group; ^#^, *P* < 0.05 compared with the morphine tolerance group; GFAP, glial fibrillary acidic protein; NC, negative control

Immunofluorescence staining was employed to detect GFAP protein expression. Compared with the control group, the GFAP protein expression of rats in the morphine tolerance, miR-365 mimic, miR-365 inhibitor and miR-365 NC groups all increased (*P* < 0.05). Compared with the morphine tolerance group, the GFAP protein expression in the miR-365 mimic group decreased (*P* < 0.05), while the GFAP protein expression in the miR-365 inhibitor group increased (*P* < 0.05); the GFAP protein expression in the miR-365 NC group showed no significant change (*P* > 0.05), as shown in Fig. 6. This led to the suggestion that miR-365 overexpression might lower GFAP protein expression, inhibiting the activation of astrocyte.

## Discussion

Morphine tolerance develops after long-term morphine use, that consequently results in the reduction of the desired analgesic effects [20]. Owing to the fact that morphine tolerance and pain share similar signaling pathways, miRs may potentially be involved in the progress of morphine tolerance [21]. The key objective of the present study was to evaluate the effects of miR-365 on morphine analgesic tolerance by targeting β-arrestin2 in a rat model of morphine tolerance. The collective results showed strong evidence that miR-365 could relieve the development of morphine analgesic tolerance to some degree by targeting β-arrestin2 through reducing the content of IL-1β, TNF-α and IL-18 and inhibiting the activation of astrocyte and the ERK/CREB signaling pathway.

The findings showed that compared with the rats in the morphine tolerance group, higher miR-365 expression and %MPE were found in the miR-365 mimic group, suggesting that miR-365 and %MPE may be related to morphine administration. A correlation was detected indicating that morphine analgesia is directly proportional to the %MPE [22]. Our study also shows that compared with rats in the morphine tolerance group, the β-arrestin2, ERK and GFAP mRNA expressions were reduced in the miR-365 mimic group in a time-dependent manner, indicating that the overexpression of miR-365 might attribute to decreased β-arrestin2 mRNA expressions [4]. It has also been demonstrated by Yang et al. who demonstrated that the reduction of β-arrestin 2 by intrathecal injection of siRNA could alleviate morphine tolerance in rats [23]. The ERK pathway plays a critical role in control of cellular responses to stress and rewarding effects of many drugs of abuse, such as nicotine, morphine and cocaine [24]. CREB has been proved to be involved in the adaptive response to drug addiction, emotional behavior and stress [25]. Moreover, as critical points of convergence, ERK and CREB have emerged in signaling pathways regulating neuronal plasticity [26, 27]. In morphine-treated animals, acute and sub-chronic stress increases the levels of p-ERK and p-CREB in the mesocorticolimbic system, showing that morphine induces the enhancement of the mentioned factors [24]. The pretreatment with ERK inhibitor notably suppressed miR-365 promoter activity, which indicated the regulatory role of ERK on the miR-365 transcription [28]. GFAP is recognized to be a marker protein for astrogliosis [29] as there is an increase in the protein during ischemic lesions due to neurodegenerative disorders. It is reported that in various neuro-inflammatory diseases, the up-regulated GFAP expression indicates the severity of astroglial activation [30]. A recent evidence suggested that activation of glial cell, especially the activation of microglia, played an important role in morphine tolerance [31]. miRs emerged as the decisive factor in microglia reactivity and served as the fine tuner of post-transcriptional events mediated neuronal gene expression [32].

Furthermore, our study demonstrated that compared with the control group, rats in the remaining four groups had elevated contents of IL-1β, TNF-α and IL-18. And compared with rats in the morphine tolerance group, lower contents of IL-1β, TNF-α and IL-18 were found in the miR-365 mimic group. The interleukins IL-1β and IL-18 belong to the pro inflammatory IL-1 cytokine superfamily, monocytes and macrophages are the major cellular sources of IL-1β and IL-18 [33]; in addition to other cells including renal tubular epithelial cells and vascular endothelial cells, may also produce these cytokines under certain conditions [34]. The activation of microglia can generate proinflammatory cytokines, such as IL-1β, which may weaken morphine analgesia efficacy and lead to morphine tolerance [35]. Previous statistics indicated that the mRNA expression of proinflammatory cytokines, IL-18 and IL-1β, down-regulated by the activation of G-protein coupled receptor 43 (GPR43) and the knockdown of β-arrestin 2, can recover the expression of the proinflammatory cytokines [36]. In inflammatory diseases, chronic TNF exposure can suppress adaptive immunity and T-cell function [37]. TNF, a proinflammatory cytokine secreted from macrophages and adipocytes, also plays a significant role in innate immunity and host defense, particularly in mycobacterial infections, and it can both enhance and suppress adaptive immunity [38]. Some data implied that β-arrestin 2 functions to negatively regulate the inflammatory response (TNF-α) in polymicrobial sepsis [39]. Previous studies showed that the mRNA expressions of TNF-α could be induced by morphine through dopamine and opioid receptors [40]. Moreover, previous studies have demonstrated that the induction of miR-365 may potentially represent a vicious gateway that results in the abnormal release of TNFα [32]. A study reports that characterized by elevated levels of endogenous IL-1, rats displayed resistance to morphine analgesia [41]. As reflected in previous study, the up-regulated expression of miR-365 could reverse the established morphine tolerance [4].

## Conclusions

In conclusion, the results obtained during this study demonstrated that miR-365 could reduce the susceptibility to morphine analgesic tolerance by targeting β-arrestin2, resulting in the reduction of the contents of IL-1β, TNF-α and IL-18 and inhibiting the activation of astrocyte and the ERK/CREB signaling pathway. However, the study did encounter certain limitations. The goal of miR overexpression is to restore the physiological levels of miR for optimal function and avoid unwanted side effects of high levels of miR-356. However, neither the lentiviral-mediated overexpression of miR-365 nor a miR-365 mimic met these requirements. In order to fully understand the specific mechanisms of miR-365 targeting of β-arrestin2, further experimental investigations, such as the investigation into the genetic deletion of miR-365 in the spinal cord, are required in order to clarify the regulatory mechanisms of miR-365. Furthermore, clinical trials would be required to further verify the findings of the study, and ascertain if said findings may be applied to human beings.
